# Early Inpatient Implementation of an ERAS-Informed Stepwise Rehabilitation Pathway After Percutaneous Intramyocardial Septal Radiofrequency Ablation for Obstructive Hypertrophic Cardiomyopathy: A Retrospective Cohort Study

**DOI:** 10.3390/jcm15187029

**Published:** 2026-09-10

**Authors:** Danyan Yang, Boren Tan, Rong Li, Qifeng Zhu, Huajun Li, Yue Mao

**Affiliations:** 1 Department of Nursing, The Second Affiliated Hospital of Zhejiang University School of Medicine, Hangzhou 310009, China; 20160809@zju.edu.cn (D.Y.);; 2 Department of Cardiology, The Second Affiliated Hospital of Zhejiang University School of Medicine, Hangzhou 310009, China; borentan02@zju.edu.cn (B.T.);; 3 State Key Laboratory of Transvascular Implantation Devices, Hangzhou 310009, China; 4 Heart Regeneration and Repair Key Laboratory of Zhejiang Province, Hangzhou 310009, China; 5 Transvascular Implantation Devices Research Institute, Hangzhou 310053, China

**Keywords:** cardiac rehabilitation, exercise therapy, enhanced recovery after surgery, hypertrophic cardiomyopathy, length of stay

## Abstract

**Background:** Percutaneous intramyocardial septal radiofrequency ablation (PIMSRA), also known as the Liwen procedure, is an emerging septal reduction therapy for obstructive hypertrophic cardiomyopathy (OHCM), but standardized early rehabilitation pathways after PIMSRA are lacking. We aimed to develop an enhanced recovery after surgery (ERAS)-informed, risk-stratified stepwise rehabilitation pathway after PIMSRA, describe its documented early inpatient implementation in selected clinically stable patients, report preliminary in-hospital safety observations, and explore its associations with hospitalization outcomes. **Methods:** This exploratory single-center retrospective cohort study included 102 patients with OHCM who underwent first-time PIMSRA between 1 July 2023 and 3 August 2025 and met the same predefined post-PIMSRA rehabilitation eligibility criteria. Patients were classified according to the care pathway actually received: usual care (*n* = 53) or early inpatient implementation of the ERAS-informed rehabilitation pathway plus usual care (*n* = 49). The planned pathway incorporated rehabilitation eligibility screening, risk stratification, staged early mobilization, structured physiological monitoring with predefined stop criteria, multidisciplinary coordination, and patient education. Pathway selection was nonrandomized and reflected patient- or family-related preferences and implementation or logistical factors. **Results:** The ERAS-informed group had a shorter ICU length of stay than the usual-care group in the primary analysis [20.0 (5.8–26.6) h vs. 23.0 (17.1–45.5) h; Hodges–Lehmann difference, −6.0 h; 95% CI, −16.51 to −0.53; *p* = 0.022]. Three ICU stays > 100 h occurred in the usual-care group and none in the ERAS-informed group; after excluding these observations, the comparison was attenuated (*p* = 0.060). The total hospital length of stay was shorter in the ERAS-informed group [8.0 (7.0–11.0) d vs. 9.0 (8.0–13.0) d; difference, −1.0 d; 95% CI, −2.02 to −0.03; *p* = 0.029], whereas the post-PIMSRA length of stay did not differ significantly (*p* = 0.058). The admission-to-PIMSRA interval was also not significantly different (*p* = 0.309). No rehabilitation-related adverse events were recorded in the ERAS-informed group during hospitalization (0/49; exact binomial 95% CI, 0.0–7.3%). **Conclusions:** Among selected clinically stable patients after PIMSRA, early inpatient implementation of pathway components was achievable, primarily during the period corresponding to planned stages 1–2. No rehabilitation-related adverse events were recorded, providing preliminary patient-level in-hospital safety observations. Fidelity, adherence, completion, and reproducibility of the complete four-stage, risk-stratified pathway were not established. Length-of-stay associations were exploratory and should not be interpreted causally.

## 1. Introduction

Hypertrophic cardiomyopathy (HCM) is the most common inherited cardiomyopathy [1]. In symptomatic obstructive HCM (OHCM), left ventricular outflow tract obstruction contributes to dyspnea, chest discomfort, syncope, heart failure progression, and impaired quality of life [2,3]. Contemporary guidelines emphasize individualized, multidisciplinary management, including pharmacotherapy, sudden-death risk assessment, exercise counseling, and referral for septal reduction therapy when symptoms and gradients remain clinically significant [2,4]. Newer pharmacologic and septal reduction therapies have expanded treatment options for symptomatic obstruction [5,6].

Percutaneous intramyocardial septal radiofrequency ablation (PIMSRA), also known as the Liwen procedure, is a minimally invasive septal reduction strategy in which radiofrequency energy is delivered into the hypertrophied interventricular septum under echocardiographic guidance [7,8]. In the present clinical setting, PIMSRA was considered for patients with symptomatic OHCM despite guideline-directed medical therapy who remained candidates for septal reduction therapy after multidisciplinary assessment [2,4,7,8]. Follow-up studies have examined gradient reduction, symptom improvement, myocardial remodeling, and myocardial mechanics after PIMSRA [9,10], and complication analyses and recent reviews also support the feasibility of this approach while emphasizing the need for careful patient selection and monitoring [11,12]. Evidence remains limited regarding standardized perioperative rehabilitation and early mobilization after PIMSRA [13].

Exercise recommendations for HCM have shifted from broad restriction toward individualized prescription and shared decision-making [2,14]. Moderate-intensity exercise and supervised cardiac rehabilitation may be feasible and beneficial in selected patients with HCM [15,16], and more recent exercise studies have further supported structured activity in carefully evaluated patients [17,18]. However, outpatient HCM exercise data cannot be directly extrapolated to the immediate post-PIMSRA period, when arrhythmia surveillance, hemodynamic stability, puncture-site management, and symptom monitoring are central safety concerns.

Enhanced recovery after surgery (ERAS) provides a framework for standardizing perioperative care, early mobilization, monitoring, and patient education [19]. In cardiac surgery, ERAS programs and early mobilization have been associated with improved recovery processes and physical function, although implementation varies across settings [20,21]. In this study, the ERAS-informed pathway primarily adapted ERAS principles related to early mobilization, structured monitoring, multidisciplinary coordination, and patient education, rather than representing a comprehensive multimodal ERAS program. For patients with OHCM undergoing PIMSRA, no widely accepted early exercise rehabilitation protocol has been established; therefore, we developed an ERAS-informed, risk-stratified stepwise early rehabilitation pathway after PIMSRA, described its documented early inpatient implementation in selected clinically stable patients, reported preliminary in-hospital safety observations, and explored associations with hospitalization outcomes.

## 2. Materials and Methods

### 2.1. Study Design, Setting and Ethics

This was an exploratory, single-center, retrospective, nonrandomized cohort study with concurrent comparison groups, conducted in a tertiary hospital and reported in accordance with the STROBE statement. The study included patients with OHCM who underwent first-time PIMSRA between 1 July 2023 and 3 August 2025, with the study period defined according to the dates of the PIMSRA procedures. The usual-care and ERAS-informed early rehabilitation pathways were implemented concurrently during the same calendar period.

No formal a priori sample size calculation was performed: the sample size was determined by the number of eligible patients with available clinical data during the study period.

The protocol and use of clinical data were reviewed and approved by the institutional human research ethics committee (approval No. (2022) Lunshenyan 0472; event acceptance No. IR2022056; review date 8 June 2022). The approval covered use and analysis of routinely collected clinical data for this cohort. The study was conducted in accordance with the Declaration of Helsinki, and written informed consent for participation was obtained as documented in the clinical records.

### 2.2. Participants and Group Classification

Eligible patients underwent first-time PIMSRA for OHCM, met predefined post-procedural rehabilitation criteria, and had sufficient clinical data for the main hospitalization outcomes. Eligibility required stable hemodynamics and oxygenation, adequate consciousness and cooperation, stable puncture-site status, and no contraindication to early mobilization. Patients with severe complications, inability to participate, or insufficient data were excluded. Because a complete contemporaneous screening log was not available in this retrospective study, the total number of patients assessed before group classification and the number of and specific reasons for exclusions could not be reliably reconstructed.

After PIMSRA, rehabilitation eligibility was assessed contemporaneously in both groups as part of routine clinical care using the same predefined criteria. This assessment was performed before initiation of any ERAS-informed rehabilitation, and eligibility in the usual-care group was not reconstructed retrospectively for the present study. Thus, all patients included in the comparative analysis had been judged clinically stable and eligible for rehabilitation at the time of care.

Among eligible patients, initiation of the ERAS-informed pathway was nonrandom and was influenced by patient or family willingness and psychological readiness, as well as implementation factors such as procedure timing, rehabilitation personnel availability, and ward workload. Patients who received the pathway were classified as the ERAS-informed group, whereas those who continued to receive routine post-procedural care were classified as the usual-care group. These factors affected pathway receipt but did not indicate failure to meet the predefined rehabilitation eligibility criteria.

The usual-care group (*n* = 53) received routine post-procedural care, including bed rest, puncture-site observation, gradual mobilization, routine education, and telephone follow-up, whereas the ERAS-informed group (*n* = 49) received routine care plus early inpatient implementation of components of the planned ERAS-informed stepwise rehabilitation pathway. Rehabilitation was initiated after confirmation of clinical stability and adjusted according to symptoms, vital signs, puncture-site status, and exercise tolerance.

### 2.3. Septal Ablation and Usual Periprocedural Care

PIMSRA candidacy was determined by a multidisciplinary HCM team. Patients underwent PIMSRA for symptomatic OHCM despite guideline-directed medical therapy and were considered candidates for septal reduction therapy on the basis of persistent symptoms, echocardiographic evidence of obstruction, and overall clinical suitability. The rehabilitation study did not prescribe or modify the indications for PIMSRA, the ablation technique, periprocedural pharmacological treatment, monitoring, ICU transfer, or hospital discharge decisions. Periprocedural pharmacological treatment was determined by the treating cardiology team according to routine clinical practice and was not specified or modified by the rehabilitation pathway. For the baseline medication comparison, pre-PIMSRA medication use was defined as medication documented as ongoing and not withheld during the 24 h preceding PIMSRA. Patient-level data on post-PIMSRA intravenous vasoactive support and intravenous diuretic therapy were not available in the source dataset.

After PIMSRA, patients received routine monitoring of hemodynamics, electrocardiographic changes, oxygen saturation, transapical puncture-site status, heart failure symptoms, and procedure-related complications. Post-procedural management of the percutaneous transapical chest-wall puncture site included a pressure dressing for 12 h; out-of-bed activity was temporarily restricted during this period, whereas in-bed limb exercises could be performed when clinically appropriate. Decisions regarding transfer from the ICU and hospital discharge were made by the treating team based on routine clinical considerations, including stable hemodynamics and oxygenation, absence of progressive arrhythmia or heart failure, stable transapical puncture-site status, completion of the required post-procedural assessment, and the ability to perform basic activity and complete discharge education. Treating teams were aware of the care pathway received by each patient. Although routine clinical criteria were considered, no prospectively standardized, study-specific criteria for transfer from the ICU or hospital discharge were applied, and these decisions were not independently adjudicated.

### 2.4. Development of the Rehabilitation Program

A multidisciplinary rehabilitation team was established before implementation, which included two cardiologists, two critical-care nursing experts, two case managers, one rehabilitation therapist, three rehabilitation specialist nurses, and one echocardiographer. The team reviewed relevant evidence from the international and Chinese literature databases, including PubMed, Wanfang, and VIP, as well as clinical practice guidelines and rehabilitation recommendations. The pathway was developed by the multidisciplinary team based on ERAS principles, HCM exercise rehabilitation evidence, PIMSRA-specific clinical considerations, and local clinical experience. The final program specified eligibility screening, rehabilitation risk stratification, staged exercise content, monitoring indicators, progression rules, outcome evaluation, and safety stop criteria.

The risk-stratification framework was applied only after rehabilitation eligibility had been confirmed. Active heart failure, sustained or malignant arrhythmia, hemodynamic instability, clinically significant oxygen desaturation, active puncture-site bleeding, or another severe complication requiring escalation of care resulted in deferral of rehabilitation and was not classified as a high-risk rehabilitation category.

Among eligible patients, low risk required all low-risk criteria, high risk was assigned when any high-risk criterion was present, and the remaining patients were classified as moderate risk. Symptoms, cardiac rhythm, left ventricular ejection fraction, and exercise capacity were considered in the classification (Table 1), while cardiopulmonary exercise testing-derived variables were used only when available. Psychological readiness was assessed after medical rehabilitation eligibility had been confirmed to evaluate understanding, willingness, and the ability to follow instructions; it was not a formal medical eligibility or risk-stratification criterion but could influence whether the ERAS-informed pathway was implemented. Although risk stratification was a planned component of the pathway, individual patient-level risk-category assignments were not systematically retained in the retrospective study dataset; therefore, fidelity to and reproducibility of risk-specific intervention delivery could not be evaluated.

### 2.5. Rehabilitation Eligibility Screening and Intervention Delivery

After PIMSRA, the same predefined rehabilitation eligibility criteria were assessed contemporaneously in both groups as part of routine clinical care. In patients who received the ERAS-informed pathway, clinical stability and exercise eligibility were reassessed before each supervised rehabilitation session. Exercise was initiated only when the resting heart rate was 50–130 beats/min, systolic blood pressure was 90–180 mmHg, oxygen saturation was ≥90%, cardiac rhythm was clinically stable, no intolerable symptoms were present at rest, and the transapical puncture site showed no active bleeding or substantial dressing separation. Patients who did not meet these session-level criteria were reassessed, and rehabilitation was deferred.

**Figure 1:**
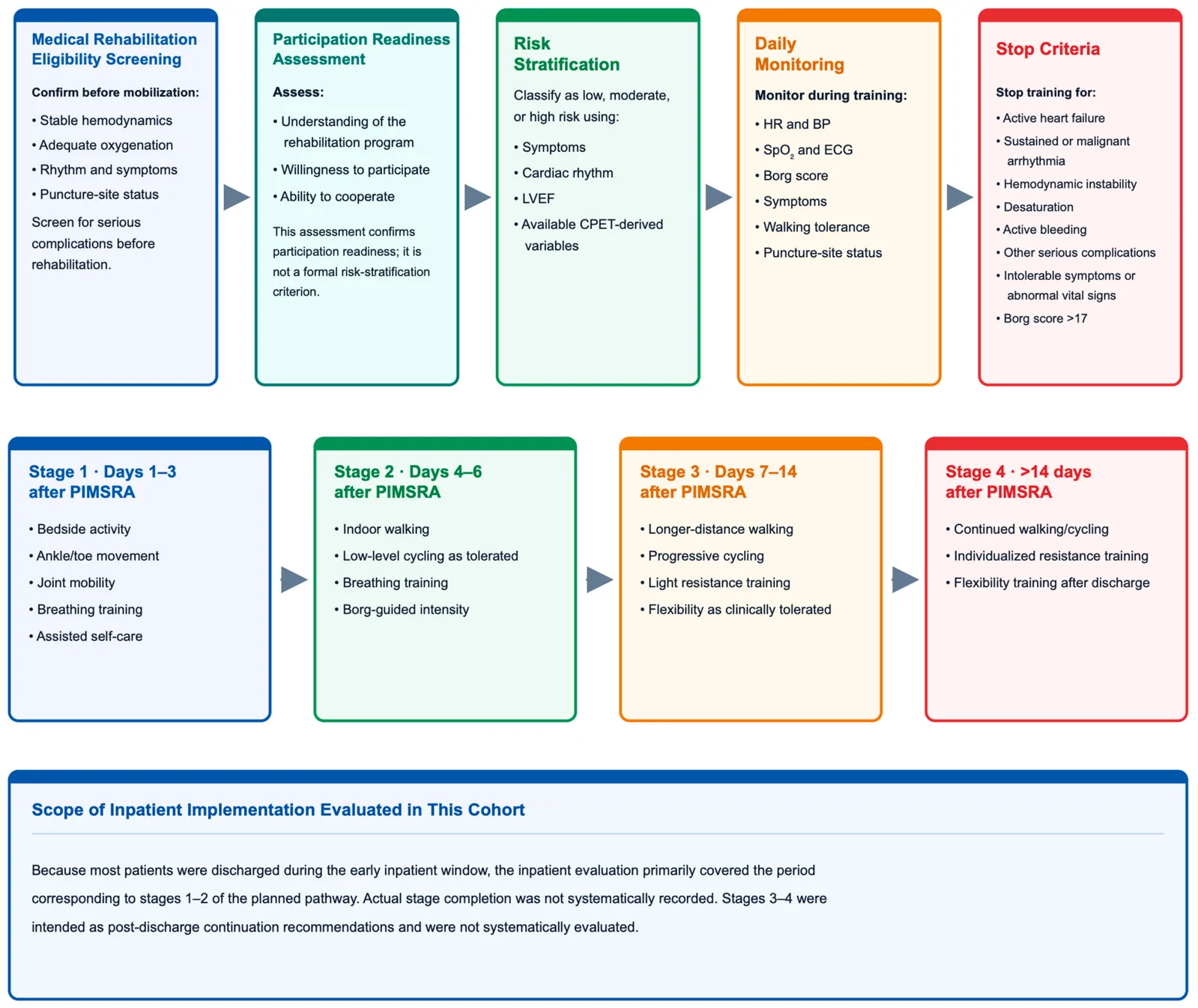
Planned ERAS-informed stepwise rehabilitation pathway after PIMSRA and the scope of early inpatient implementation evaluated in this cohort.

The planned rehabilitation pathway specified four stages: stage 1—in-bed and bedside activity during post-procedural days 1 to 3; stage 2—indoor activity and low-level exercise during post-procedural days 4 to 6; stage 3—moderate-level exercise during post-procedural days 7 to 14; stage 4—higher-level exercise after post-procedural day 14. Content included ankle and toe movement, joint activity, diaphragmatic breathing, bedside sitting and standing, indoor walking, cycling, elastic-band resistance exercise, and flexibility training; duration, frequency, and Borg intensity differed by risk level and stage (Table 2; Figure 1).

The responsible nurse demonstrated each exercise, confirmed understanding, assessed tolerance, and reinforced progression rules. Because the median post-PIMSRA length of stay was approximately 5–6 days, the inpatient observation window primarily corresponded to stages 1–2 of the planned pathway; stages 3–4 were intended as post-discharge continuation recommendations and were not systematically evaluated in this study. Actual stage completion was also not systematically recorded.

### 2.6. Monitoring, Stop Criteria, and Adverse Events

During exercise sessions, heart rate, rhythm, oxygen saturation, blood pressure, Borg score, symptoms, and puncture-site status were monitored. Electrocardiographic and oxygen-saturation monitoring continued throughout training during the early period after PIMSRA. Training was stopped for the day if any of the following occurred: heart rate greater than 130 beats/min or lower than 50 beats/min; heart rate increase greater than 20 beats/min above baseline; oxygen saturation below 90%; systolic blood pressure greater than 180 mmHg or lower than 90 mmHg; severe arrhythmia; obvious chest distress, dyspnea, dizziness, nausea, cold sweating, or other intolerable symptoms; puncture-site bleeding or substantial dressing separation; Borg score greater than 17.

Rehabilitation-related adverse events were defined as clinically significant events occurring during or shortly after supervised rehabilitation and judged by the clinical team to be related to rehabilitation, including arrhythmia requiring treatment or training termination, symptomatic hypo- or hypertension, syncope or presyncope, worsening heart failure symptoms, oxygen desaturation requiring intervention, fall or injury, puncture-site bleeding, unplanned transfer back to the ICU, or death. Post-PIMSRA adverse events were reviewed separately and were not classified as rehabilitation-related unless a temporal and clinical relationship to rehabilitation was documented. Attribution was based on retrospective clinical documentation and clinical-team judgment rather than independent adjudication.

### 2.7. Outcome Measures

The primary care-process outcome was ICU length of stay, recorded in hours, and the primary safety outcome was the number of patients with at least one recorded rehabilitation-related adverse event in the ERAS-informed group during hospitalization. Secondary exploratory outcomes included post-PIMSRA length of stay, total hospital length of stay, echocardiographic parameters, and NYHA functional class. Post-PIMSRA and hospital lengths of stay were defined as the period from the PIMSRA procedure and hospital admission to hospital discharge, respectively, while the admission-to-PIMSRA interval was defined as the number of days from hospital admission to the PIMSRA procedure.

Baseline clinical variables included body surface area, systolic and diastolic blood pressure, and heart rate. Echocardiographic variables assessed at baseline and discharge included interventricular septal thickness at end-diastole (IVSd, cm), left ventricular end-diastolic dimension (LVEDd, cm), left ventricular posterior wall thickness at end-diastole (LVPWd, cm), left atrial diameter (LA, cm), left ventricular ejection fraction (LVEF, %), pulmonary artery systolic pressure (PASP, mmHg), and valvular regurgitation grade. Additional baseline-only variables were maximum left ventricular wall thickness and maximum left ventricular outflow tract pressure gradient (LVOT-PGmax). The measurement condition for the latter was not consistently documented; therefore, separate resting and provoked gradients could not be reported.

Functional outcome assessment was limited to NYHA functional class recorded before the intervention and at discharge.

### 2.8. Data Management and Quality Control

Team members received standardized training on rehabilitation content, monitoring indicators, emergency management, and data recording. Data were extracted from the clinical dataset and checked by two staff members, and discrepancies or missing entries were reviewed against clinical records when possible. No imputation was performed; analyses used available cases.

### 2.9. Statistical Analysis

Continuous variables were expressed as mean ± standard deviation (SD) or median (interquartile range, IQR), and categorical variables as counts and percentages. Between-group comparisons used the independent-samples *t* test or Mann–Whitney U test for continuous variables, and the chi-square test or Fisher’s exact test for categorical variables. Within-group pre–post comparisons used paired *t* tests or Wilcoxon signed-rank tests as appropriate.

Echocardiographic change was calculated as discharge minus baseline, and between-group differences in change were calculated as ERAS-informed minus usual care and tested using Welch independent-samples *t* tests. Pulmonary artery systolic pressure analyses used available paired data.

Length-of-stay outcomes were compared using Mann–Whitney U tests, with Hodges–Lehmann estimates and 95% confidence intervals (CIs).

Exploratory post hoc multivariable linear regression models of log (LOS + 1) were adjusted for care pathway, age, sex, baseline NYHA class, IVSd, and calendar time. Complete-case analysis included 101 patients per model. The exponentiated care-pathway coefficient, exp(β), was interpreted as the adjusted geometric-mean ratio of LOS + 1 for ERAS-informed vs. usual care.

Model diagnostics included variance inflation factors, residual plots, Q–Q plots, externally studentized residuals, and Cook’s distance. Sensitivity analyses excluded ICU stays >100 h or total hospital stays > 30 days. The admission-to-PIMSRA interval was also compared between groups. For the zero-event safety outcome, an exact Clopper–Pearson 95% CI was calculated.

Echocardiographic and regression analyses were exploratory, with no adjustment for multiple comparisons, and were performed using SPSS version 26.0 and R version 4.4.3.

## 3. Results

### 3.1. Study Population and Baseline Characteristics

A total of 102 patients were included in the final analysis, including 53 in the usual-care group and 49 in the ERAS-informed group, and hospitalization and NYHA outcomes were available for all included patients.

Baseline characteristics are summarized in Table 3. The mean age was 59.0 ± 13.3 and 56.3 ± 12.9 years in the usual-care and ERAS-informed groups (*p* = 0.287), respectively, and approximately half of the patients in each group were female (50.9% vs. 51.0%, *p* = 1.000). Most patients were classified as NYHA class II at baseline. Available pre-PIMSRA medication use is summarized in Table 3; β-blockers were the most frequently recorded medication class in both groups, used in 48 of 53 (90.6%) and 46 of 49 patients (93.9%) in the usual-care and ERAS-informed groups, respectively. Absolute standardized differences across the recorded medication categories ranged from 0.01 to 0.19, indicating no large imbalance in the recorded medication classes. However, these data did not capture medication dose, treatment duration, periprocedural treatment changes, or post-PIMSRA intravenous pharmacological therapy.

Absolute standardized differences were largest for IVSd (0.50), LVPWd (0.45), heart rate (0.40), tricuspid regurgitation ≥ 2+ (0.35), body surface area (0.33), and atrial fibrillation/flutter (0.33), while maximum LV wall thickness showed only a small between-group difference (absolute standardized difference, 0.17; *p* = 0.389). Heart rate was lower (72.0 ± 12.4 bpm vs. 76.9 ± 12.4 bpm; *p* = 0.051) and IVSd, LVPWd, and maximum LV wall thickness were greater in the ERAS-informed group than in the usual-care group (IVSd: 1.99 ± 0.61 cm vs. 1.72 ± 0.46 cm, *p* = 0.014; LVPWd: 1.33 ± 0.59 cm vs. 1.13 ± 0.18 cm, *p* = 0.031; maximum LV wall thickness: 2.34 ± 0.52 cm vs. 2.25 ± 0.53 cm, *p* = 0.389). Other recorded echocardiographic parameters, including LVEDd, LA diameter, LVEF, PASP, and LVOT-PGmax, were not significantly different between groups. These findings indicate baseline imbalance in selected clinical and wall-thickness measures.

### 3.2. Hospitalization Outcomes

**Figure 2:**
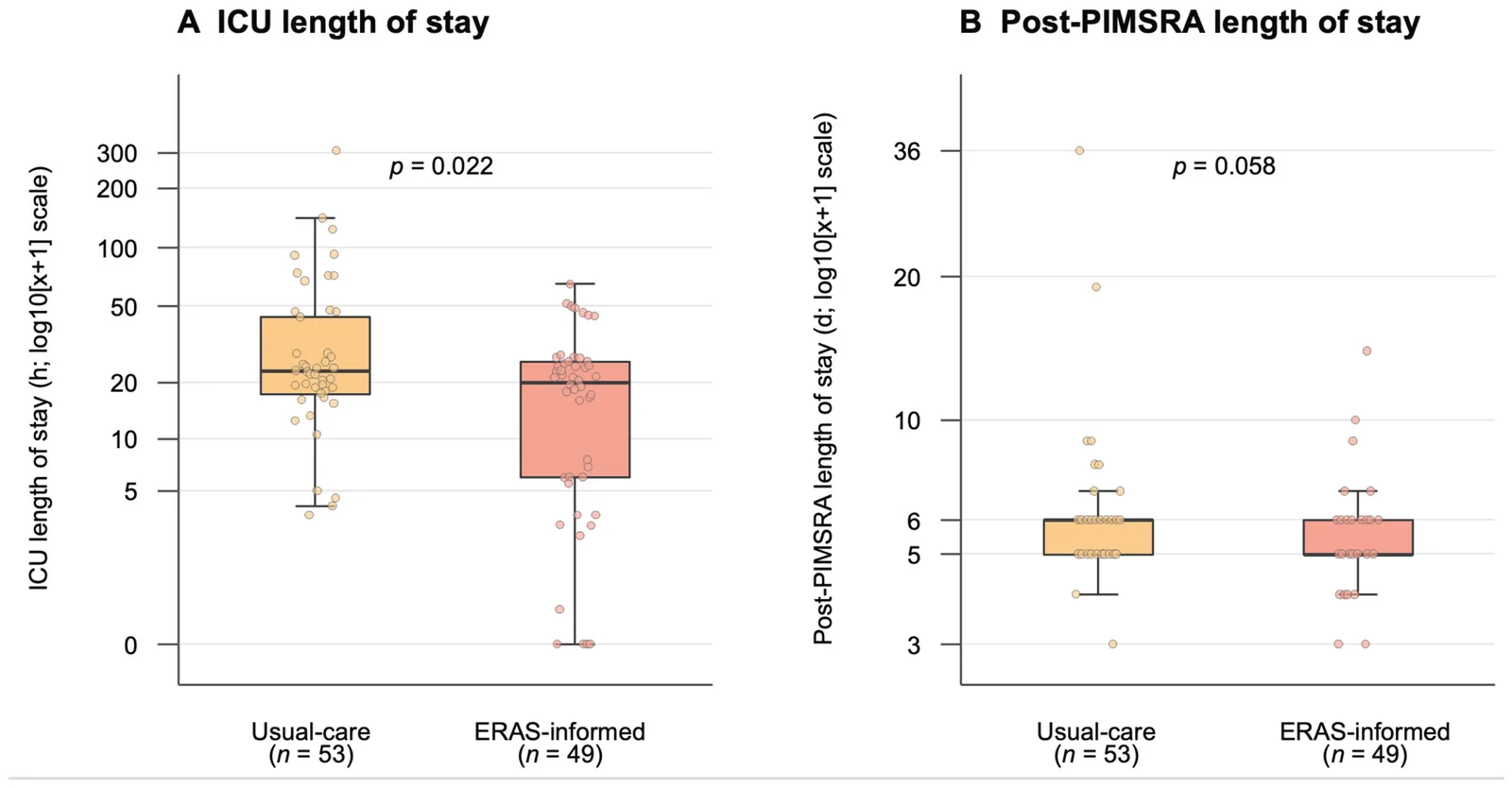
ICU and post-PIMSRA lengths of stay.

Hospitalization outcomes are summarized in Table 4, and the distributions of ICU and post-PIMSRA lengths of stay are shown in Figure 2. ICU length of stay was right-skewed, particularly in the usual-care group. In the primary unadjusted analysis, median ICU length of stay was shorter in the ERAS-informed group than in the usual-care group [20.0 (5.8–26.6) h vs. 23.0 (17.1–45.5) h; Hodges–Lehmann difference, −6.0 h; 95% CI, −16.51 to −0.53; *p* = 0.022]. Because ICU transfer decisions were made by treating teams who were aware of the care pathway received and were not based on prospectively standardized, independently adjudicated study criteria, this finding should be interpreted as an exploratory care-process association rather than independent evidence of earlier clinical readiness for ICU transfer.

Three patients had ICU stays >100 h, all in the usual-care group (3/53 vs. 0/49), with stays of 123.0, 140.5, and 310.0 h. In a post hoc unadjusted sensitivity analysis excluding these observations, the between-group comparison was attenuated and no longer met the conventional threshold for statistical significance (*p* = 0.060). Thus, the primary unadjusted ICU finding was sensitive to a small number of prolonged stays.

Post-PIMSRA length of stay was numerically shorter in the ERAS-informed group but did not reach statistical significance [5.0 (5.0–6.0) d vs. 6.0 (5.0–6.0) d; Hodges–Lehmann difference, 0.0 d; 95% CI, −1.04 to 0.01; *p* = 0.058], and total hospital length of stay was also shorter in that group [8.0 (7.0–11.0) d vs. 9.0 (8.0–13.0) d; Hodges–Lehmann difference, −1.0 d; 95% CI, −2.02 to −0.03; *p* = 0.029]. The admission-to-PIMSRA interval did not differ significantly between groups [3.0 (2.0–5.0) d vs. 3.0 (2.0–7.0) d; Hodges–Lehmann difference, 0.0 d; 95% CI, −1.02 to 1.01; *p* = 0.309]. Nevertheless, total hospital length of stay includes a pre-procedural component that could not have been influenced by post-PIMSRA rehabilitation.

One patient had a total hospital stay >30 d. After excluding this observation in a post hoc unadjusted sensitivity analysis, the between-group comparison of total hospital length of stay remained statistically significant (*p* = 0.041). Both length-of-stay sensitivity analyses were post hoc and unadjusted.

In exploratory post hoc multivariable models adjusted for age, sex, baseline NYHA class, IVSd, and calendar time, exp(β) for the ERAS-informed group was 0.51 (95% CI, 0.34–0.76; *p* = 0.001), 0.88 (95% CI, 0.79–0.99; *p* = 0.029), and 0.83 (95% CI, 0.72–0.95; *p* = 0.006) for ICU, post-PIMSRA, and total hospital lengths of stay, respectively (Table 5). These exponentiated coefficients represent ratios of the adjusted geometric means of LOS + 1 rather than ratios of the arithmetic means or medians of the original outcomes. The models were not adjusted for multiple comparisons and were interpreted as hypothesis-generating. Although they adjusted for measured baseline covariates, care pathway was modeled as a fixed binary group indicator. Because the exact timing of pathway initiation—particularly the first supervised rehabilitation session relative to ICU transfer—was unavailable, the models could not account for potential time-dependent selection or reverse causation and did not establish causal effects.

### 3.3. Echocardiographic Outcomes

Baseline and discharge echocardiographic data are summarized in Table 6. In the usual-care group, IVSd, LVEDd, LVPWd, and LA diameter did not change significantly from baseline to discharge; in the ERAS-informed group, LVEDd increased from 4.14 ± 0.58 cm to 4.32 ± 0.57 cm (*p* = 0.031), whereas IVSd, LVPWd, and LA diameter did not show statistically significant within-group changes. Between-group differences in mean change, with 95% confidence intervals, are reported in Table 6. The estimated differences in change were −0.08 cm for IVSd, +0.20 cm for LVEDd, −0.16 cm for LVPWd, −0.15 cm for LA diameter, +1.11 percentage points for LVEF, and +1.38 mmHg for PASP, expressed as the ERAS-informed group minus the usual-care group. None of the corresponding 95% confidence intervals excluded zero. Because these analyses were exploratory and no multiplicity adjustment was applied, the estimates should be interpreted cautiously and not as evidence of equivalence.

LVEF decreased modestly but significantly from baseline to discharge in both groups: it fell from 68.3 ± 6.5% to 64.0 ± 6.1% (*p* < 0.001) and from 67.7 ± 6.5% to 64.5 ± 5.5% (*p* = 0.015) in the usual-care and ERAS-informed groups, respectively. The between-group difference in LVEF change was not statistically significant (*p* = 0.506). PASP tended to decrease in both groups, but within- and between-group differences were not statistically significant; this analysis should be interpreted cautiously because paired PASP values were missing in a substantial proportion of patients.

### 3.4. Functional Class, Rehabilitation Implementation, and Safety

**Figure 3:**
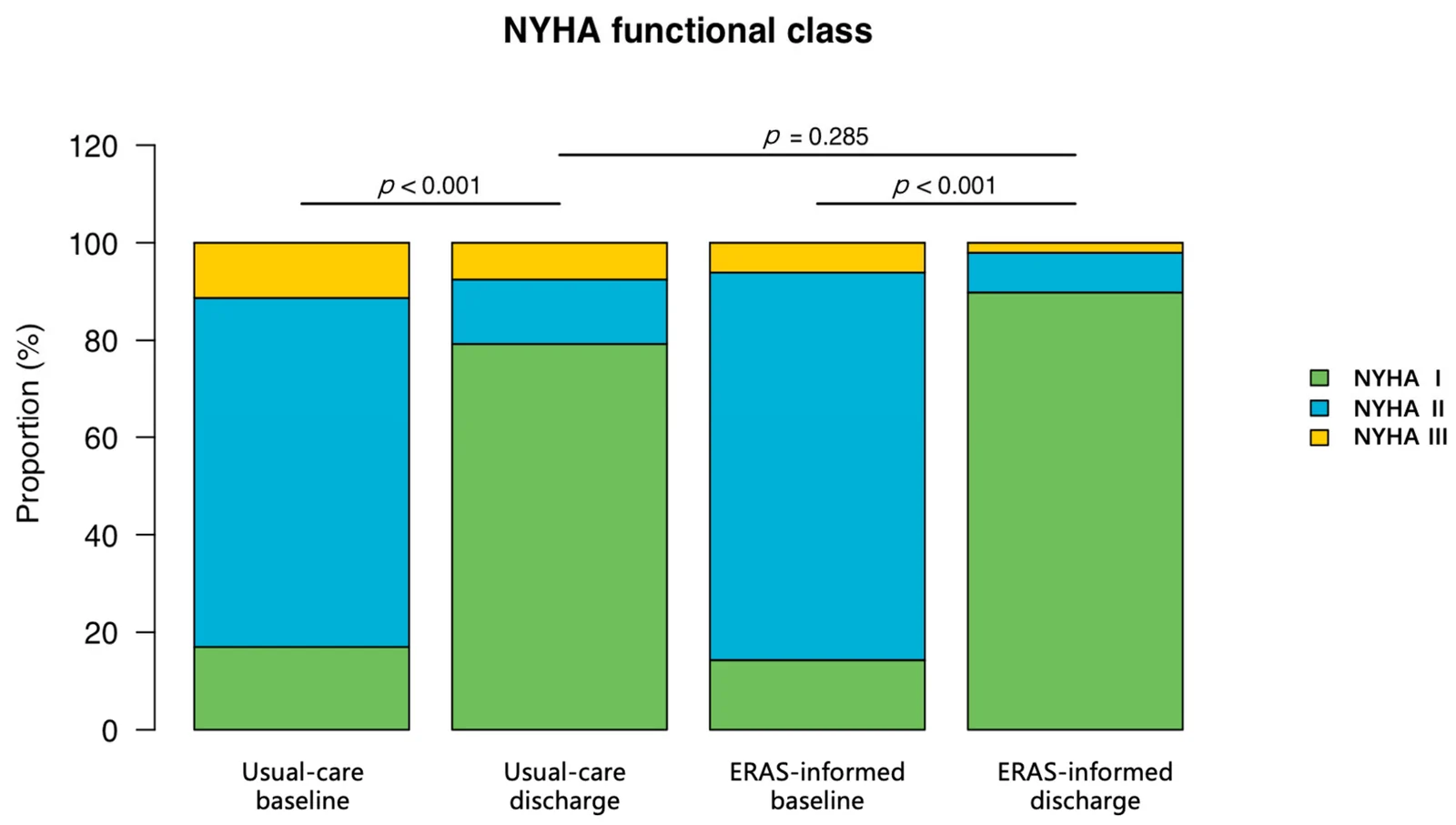
NYHA functional class at baseline and discharge in the usual-care and ERAS-informed groups.

NYHA functional class also improved substantially by discharge: the proportion of patients in NYHA class I increased from 17.0% at baseline to 79.2% at discharge and from 14.3% to 89.8% in the usual-care and ERAS-informed groups, respectively. Within-group improvement in NYHA class was significant in both groups (both *p* < 0.001). Although the ERAS-informed group had a numerically higher proportion of NYHA class I patients at discharge, the between-group distribution of discharge NYHA class was not statistically significant (*p* = 0.285); see Figure 3.

Available clinical records documented early inpatient implementation of components of the planned ERAS-informed pathway after post-PIMSRA clinical stability had been confirmed. Because the median post-PIMSRA length of stay was approximately 5–6 days, the inpatient evaluation primarily covered the period corresponding to planned stages 1–2. Stages 3–4 were intended as post-discharge continuation recommendations and were not systematically evaluated. The timing and number of supervised sessions, actual stage completion, training interruptions, patient-training days, post-discharge adherence, and individual risk-category assignments were not systematically retained. Therefore, intervention fidelity, adherence, completion, and reproducibility of risk-stratified delivery could not be evaluated.

No rehabilitation-related adverse events were recorded among the 49 patients who received the ERAS-informed pathway during hospitalization (0/49 patients; exact binomial 95% CI, 0.0–7.3%). Specifically, no event occurring during or shortly after supervised rehabilitation was documented as being related to arrhythmia requiring treatment or training termination, symptomatic hypo- or hypertension, syncope or presyncope, worsening heart failure symptoms, oxygen desaturation requiring intervention, fall or injury, puncture-site bleeding, unplanned transfer back to the ICU, or death. This estimate refers only to events judged to be related to rehabilitation and does not indicate the absence of all post-PIMSRA adverse events. Because patients with severe post-PIMSRA complications were not eligible for early rehabilitation and the number of rehabilitation sessions and patient-training days was not systematically recorded, these findings represent preliminary patient-level in-hospital safety observations rather than definitive evidence of safety. Event rates per rehabilitation session or patient-training day could not be calculated.

## 4. Discussion

This study provides an early structured description of post-PIMSRA rehabilitation for OHCM. Available clinical records documented early inpatient implementation of components of the planned ERAS-informed pathway in selected clinically stable patients, primarily during the period corresponding to planned stages 1–2. Because the timing and number of supervised sessions, actual stage completion, training interruptions, post-discharge adherence, and individual risk-category assignments were not systematically retained, the study did not establish intervention fidelity, adherence, completion, or reproducibility of the complete four-stage, risk-stratified pathway. No rehabilitation-related adverse events were recorded during hospitalization, and exploratory between-group differences in ICU and total hospital lengths of stay were observed. These findings complement PIMSRA studies that have mainly emphasized procedural feasibility, gradient reduction, remodeling, and complications rather than nursing-led early mobilization or rehabilitation implementation [8,9].

PIMSRA differs from surgical myectomy and alcohol septal ablation in procedural invasiveness, tissue injury pattern, and monitoring needs [7,8]. Accordingly, post-PIMSRA rehabilitation should not be directly extrapolated from rehabilitation protocols used after general cardiac surgery or in standard outpatient cardiac rehabilitation [19,21]. The pathway developed here integrates procedure-specific screening, risk stratification, stage-based exercise dosing, monitoring, and stop rules within a multidisciplinary nursing practice framework [21,22].

Although ICU and total hospital lengths of stay were shorter in the ERAS-informed group in the primary analyses, these findings should not be interpreted as causal effects of the rehabilitation pathway. Care-pathway selection was nonrandomized and may have been influenced by patient or family willingness, psychological readiness, procedure timing, personnel availability, ward workload, and other unmeasured factors. Moreover, because the exact timing of the first supervised rehabilitation session relative to ICU transfer was unavailable, it could not be established that rehabilitation exposure preceded ICU transfer in all patients. Patients with a more rapid uncomplicated recovery or earlier ICU transfer may have been more likely to receive the ERAS-informed pathway, rather than the pathway necessarily leading to the shorter ICU stay. In addition, ICU transfer and hospital discharge decisions were made by treating teams who were aware of the pathway received, without prospectively standardized or independently adjudicated criteria. The ICU comparison was also sensitive to three prolonged stays (> 100 h), all in the usual-care group; after their exclusion, the between-group difference was attenuated to *p* = 0.060. Although the admission-to-PIMSRA interval did not differ significantly between groups, total hospital length of stay included a pre-procedural component outside the rehabilitation exposure period. The exploratory adjusted models addressed measured baseline covariates but could not overcome uncertainty regarding rehabilitation-initiation timing or the resulting potential for time-dependent selection and reverse causation. Accordingly, the length-of-stay findings should be regarded as hypothesis-generating observational associations. Future prospective studies should use standardized ICU transfer and hospital discharge criteria.

No rehabilitation-related adverse events were recorded among the 49 patients who received the ERAS-informed pathway (0/49; exact binomial 95% CI, 0.0–7.3%). However, this finding should be interpreted cautiously because rehabilitation was implemented only in selected clinically stable patients, patients with severe post-PIMSRA complications that precluded exercise were excluded, event ascertainment and attribution were retrospective, and the numbers of supervised sessions and patient-training days were unavailable. Accordingly, these findings provide preliminary patient-level in-hospital safety observations and cannot establish definitive safety, quantify a session-level event rate, or be generalized to clinically unstable or higher-risk patients. The pathway incorporated electrocardiographic and oxygen-saturation monitoring, blood-pressure and symptom surveillance, Borg-guided intensity, puncture-site assessment, individualized progression, and predefined stop criteria. These measures should be regarded as safeguards within this selected population rather than evidence that the pathway is universally safe; they are consistent with the need for individualized exercise prescription in HCM [14,17], and are particularly relevant after PIMSRA because of the potential for arrhythmia, hypotension, residual obstruction, myocardial functional change, or puncture-site bleeding [11].

Short-term echocardiographic findings should be interpreted cautiously. LVEF decreased from baseline to discharge in both groups, but the estimated between-group difference in LVEF change was small and its 95% confidence interval included zero and was compatible with effects in both directions. Because no equivalence or noninferiority margin was prespecified, the nonsignificant comparison cannot establish equivalence or exclude a potentially adverse effect. All echocardiographic analyses were exploratory and were not adjusted for multiple comparisons. No significant between-group differences were observed in discharge changes in IVSd, LVEDd, LVPWd, LA diameter, LVEF, or PASP.

NYHA class improved in both groups, but the between-group distribution at discharge was not significant. Because no significant between-group difference was observed, these within-group changes should not be interpreted as evidence of functional efficacy of the ERAS-informed pathway. Objective endpoints such as 6-min walk distance, cardiopulmonary exercise testing, daily step counts, and validated quality-of-life measures are needed to detect more subtle functional effects [23,24].

The present study was not designed to identify treatment-effect heterogeneity or determine which patient subgroup derives the greatest benefit. Based on the eligibility criteria and the intervention actually delivered, the pathway may be most applicable to selected patients after PIMSRA who have stable hemodynamics and oxygenation, are able to cooperate with rehabilitation, have a stable transapical puncture site, and have no severe complications or contraindications to early mobilization. For these patients, particularly those requiring a structured transition from bed rest to supervised bedside and low-intensity activity, the pathway may provide a standardized framework for eligibility screening, physiological monitoring, individualized progression, and predefined stop criteria. Patients with unstable clinical conditions or severe post-PIMSRA complications require individualized reassessment before rehabilitation initiation. The inpatient evaluation primarily covered the period corresponding to stages 1–2 of the planned pathway, and no prespecified subgroup analyses or objective functional outcomes were available. Therefore, the present study cannot determine which clinical or risk subgroup derives greater benefit, and the later-stage components require prospective evaluation.

This study has several limitations. Firstly, it was a single-center, retrospective, and nonrandomized cohort study. Moreover, although all patients included in the comparative analysis met the same predefined rehabilitation eligibility criteria and the two care pathways were implemented concurrently, pathway selection was not randomized. Differences in patient preference, clinician decision-making, clinical workflow, rehabilitation resource availability, or other unmeasured factors may therefore have introduced selection bias and residual confounding. In addition, the absence of a complete screening log prevented reconstruction of the total number of patients assessed before group classification and the number of and reasons for exclusions, limiting evaluation of the selection process and the generalizability of the study population.

Although rehabilitation eligibility was assessed contemporaneously in both groups, the exact interval from PIMSRA to the eligibility assessment and, in the ERAS-informed group, to the first supervised rehabilitation session was not systematically recorded. Consequently, the temporal sequence between the first rehabilitation exposure and ICU transfer could not be established in all patients. Patients with a more rapid uncomplicated recovery or earlier ICU transfer may have been more likely to receive the ERAS-informed pathway, rather than the pathway necessarily causing the shorter ICU stay. Therefore, time-dependent selection and reverse causation remain possible when interpreting the length-of-stay findings. Concurrent implementation reduced the likelihood of a simple historical-control effect, but residual calendar-time effects related to changes in ICU practice, operator experience, or institutional care processes may still have been present.

Intervention-fidelity data were incomplete, and the safety analysis was also limited by the selected nature of the exposed population, retrospective event ascertainment, clinical-team attribution without independent adjudication, and the absence of session-level exposure denominators. Because patients with severe post-PIMSRA complications were not eligible for early rehabilitation, the absence of recorded rehabilitation-related adverse events cannot be generalized to unstable or higher-risk patients. Furthermore, detailed information regarding total supervised sessions, patient-training days, actual stage completion, walking distance, Borg-score distributions, training interruptions, and post-discharge adherence was not systematically collected. Therefore, the present findings support only documented early inpatient implementation in selected clinically stable patients, primarily during the period corresponding to planned stages 1–2. They do not establish feasibility, intervention fidelity, adherence, completion, or reproducibility of the complete four-stage pathway. In addition, because individual risk-category assignments were not systematically retained, fidelity to and reproducibility of risk-stratified intervention delivery could not be evaluated.

Furthermore, formal standardized ICU and hospital discharge adjudication criteria were not prospectively documented. Although peak LVOT gradient was available for baseline characterization, the source dataset did not distinguish whether the measurement was obtained at rest or during provocation; therefore, baseline obstructive severity and mechanism could not be characterized as comprehensively as in a prospective HCM assessment. In addition, systolic anterior motion, ablation dose, biomarker response, detailed operator-level data, and other procedural characteristics were not consistently available. Although major pre-PIMSRA medication classes were available, medication dose, duration, periprocedural treatment changes, and other concomitant pharmacological management could not be fully assessed. Patient-level data on post-PIMSRA intravenous vasoactive support and intravenous diuretic therapy were unavailable, limiting the evaluation of between-group differences in post-procedural medical treatment and the potential for residual confounding. Lastly, functional assessment was limited to NYHA class.

## 5. Conclusions

In selected clinically stable patients with OHCM after PIMSRA, early inpatient implementation of components of the planned ERAS-informed rehabilitation pathway was achievable, with documentation primarily covering the period corresponding to planned stages 1–2. The available data did not establish fidelity, adherence, completion, or reproducibility of the complete four-stage, risk-stratified pathway. No rehabilitation-related adverse events were recorded during hospitalization, providing preliminary patient-level in-hospital safety observations. Associations with ICU and total hospital lengths of stay were exploratory and should not be interpreted causally.

## Figures and Tables

**Table 1:** Risk-stratification criteria for rehabilitation exercise.

Item	Low Risk	Moderate Risk	High Risk
Symptoms during prescribed activity	No chest distress or dyspnea at rest or during prescribed low-level activity	Transient chest discomfort or dyspnea occurring only during moderate-level activity, resolving with rest, and not meeting any stop criteria	Chest distress or dyspnea occurring during low-level activity, resolving with rest, and not meeting any stop criteria
Cardiac rhythm	No clinically significant arrhythmia or complex ventricular ectopy	Stable atrial fibrillation/flutter or isolated premature beats without symptoms, hemodynamic compromise, or need for acute treatment	Ventricular couplets, triplets, or nonsustained ventricular tachycardia without hemodynamic compromise or need for acute treatment
LVEF	≥50%	40–49%	<40%
Peak oxygen uptake, mL·kg^−1^·min^−1^	≥20	15–19	<15
Predicted peak oxygen uptake, when available	≥80%	65–79%	<65%
Anaerobic threshold, mL·kg^−1^·min^−1^	≥15	12–14	<12

Note: Risk stratification was performed only after the patient had passed the rehabilitation eligibility screen. Symptoms at rest, persistent or intolerable symptoms, sustained or malignant arrhythmia, arrhythmia requiring acute treatment, hemodynamic instability, active heart failure, clinically significant oxygen desaturation, active puncture-site bleeding, or another severe complication requiring escalation of care resulted in deferral or suspension of rehabilitation rather than classification as high risk. Cardiopulmonary exercise testing-derived criteria were applied only when available. LVEF, left ventricular ejection fraction.

**Table 2:** Planned early stepwise exercise rehabilitation program after PIMSRA.

Stage	Low Risk	Moderate Risk	High Risk
Stage 1: early in-bed and bedside activity (days 1–3 after PIMSRA)	Daily activity: gradual transition to toileting, washing, and eating after the initial 12 h puncture-site observation period and confirmation of clinical stability. Exercise: active ankle dorsiflexion and plantar flexion for 10–15 min, 2–3 times/day; gradual sitting and standing at bedside. Breathing: diaphragmatic breathing for 15–20 min, 1–2 times/day.	Daily activity: assisted self-care after the initial 12 h puncture-site observation period and confirmation of clinical stability. Exercise: passive or active ankle/toe movement and joint activity for 5–10 min, 2–3 times/day; gradual sitting and standing at bedside. Breathing: diaphragmatic breathing for 10–15 min, 1–2 times/day.	Daily activity: assisted self-care after the initial 12 h puncture-site observation period and confirmation of clinical stability. Exercise: passive or active ankle/toe movement and joint activity for 3–5 min, 1–2 times/day; gradual sitting and standing at bedside. Breathing: diaphragmatic breathing for 5–10 min, 1–2 times/day.
Stage 2: indoor activity and low-level exercise (days 4–6 after PIMSRA)	Indoor walking for 10–15 min, gradually up to 30 min, 1–2 times/day; unloaded cycling for 10–15 min, twice/day; Borg score 11–12; elastic-band stretching 2–5 min as tolerated; diaphragmatic breathing for 20–30 min, twice/day.	Slow indoor walking for 10–15 min, 1–2 times/day; unloaded cycling for 5–10 min, 1–2 times/day if tolerated; Borg score 9–10; no resistance training; diaphragmatic breathing for 15–20 min, twice/day.	Slow indoor walking for 5–10 min once/day; unloaded cycling for 3–5 min once/day if tolerated; Borg score 6–8; no resistance training; diaphragmatic breathing for 5–10 min, twice/day.
Stage 3: moderate-level exercise (days 7–14 after PIMSRA)	Indoor walking for 30 min, twice/day; progressive cycling for 15–20 min, twice/day; Borg score 13–14; elastic-band resistance exercise for 10–15 min; flexibility training for shoulders, waist, and legs with 6–15 s stretching, 3–5 repetitions; diaphragmatic breathing 20–30 min, 3 times/day.	Indoor walking for 30 min, 1–2 times/day; cycling for 10–15 min, twice/day; Borg score 11–12; elastic-band stretching for 5–10 min as tolerated; flexibility training as tolerated; diaphragmatic breathing for 15–20 min, 3 times/day.	Indoor walking for 30 min, 1–2 times/day; cycling for 10–15 min, twice/day; Borg score 9–10; elastic-band stretching for 2–5 min as tolerated; flexibility training as tolerated; diaphragmatic breathing for 10–15 min, 2–3 times/day.
Stage 4: higher-level exercise (>14 days after PIMSRA)	Indoor walking for 30 min, twice/day; progressive cycling for 20–30 min, twice/day; Borg score 15–16; resistance training for about 15 min as tolerated; flexibility training with 30–90 s stretching, 3–5 repetitions; diaphragmatic breathing for 20–30 min, 3 times/day.	Indoor walking for 30 min, twice/day; progressive cycling for 15–20 min, twice/day; Borg score 13–14; resistance training for about 10 min as tolerated; flexibility training with 6–15 s stretching, 3–5 repetitions; diaphragmatic breathing for 20–30 min, 3 times/day.	Indoor walking for 30 min, twice/day; progressive cycling for 10–15 min, twice/day; Borg score 11–12; resistance training for 5–10 min as tolerated; flexibility training with 6–15 s stretching, 3–5 repetitions; diaphragmatic breathing for 15–20 min, 3 times/day.

PIMSRA, percutaneous intramyocardial septal radiofrequency ablation. Stage duration was adjusted dynamically according to individual clinical status. Stages 3–4 were intended as post-discharge continuation recommendations and were not systematically evaluated in this cohort. Actual stage completion and adherence to risk-specific exercise prescriptions were not systematically recorded.

**Table 3:** Baseline characteristics of the study population.

Variable	Usual-Care Group (*n* = 53)	ERAS-Informed Group (*n* = 49)	*p* Value	Absolute Standardized Difference
Age, years	59.0 ± 13.3	56.3 ± 12.9	0.287	0.21
Female sex, *n* (%)	27 (50.9)	25 (51.0)	1.000	0.00
Body mass index, kg/m^2^	24.7 ± 3.2	24.6 ± 3.0	0.824	0.04
Body surface area, m^2^	1.66 ± 0.21	1.72 ± 0.15	0.097	0.33
Systolic blood pressure, mmHg	127.0 ± 19.4	128.2 ± 18.9	0.769	0.06
Diastolic blood pressure, mmHg	74.1 ± 12.3	74.1 ± 11.1	0.995	0.00
Heart rate, bpm	76.9 ± 12.4	72.0 ± 12.4	0.051	0.40
NYHA class, *n* (%)			0.575	0.21
Class I	9 (17.0)	7 (14.3)		
Class II	38 (71.7)	39 (79.6)		
Class III	6 (11.3)	3 (6.1)		
Smoking history, *n* (%)	13 (24.5)	10 (20.4)	0.640	0.11
Hypertension, *n* (%)	21 (39.6)	22 (44.9)	0.691	0.09
Diabetes mellitus, *n* (%)	5 (9.4)	3 (6.1)	0.716	0.13
Atrial fibrillation/flutter, *n* (%)	5 (9.4)	1 (2.0)	0.206	0.33
Syncope history, *n* (%)	5 (9.4)	3 (6.1)	0.716	0.13
PCI history, *n* (%)	3 (5.7)	2 (4.1)	1.000	0.08
Stroke history, *n* (%)	0 (0.0)	1 (2.0)	0.485	0.20
Current malignancy, *n* (%)	0 (0.0)	1 (2.0)	0.485	0.20
Aortic regurgitation ≥2 +, *n* (%)	1 (1.9)	2 (4.1)	0.607	0.13
Mitral regurgitation ≥ 2+, *n* (%)	11 (20.8)	11 (22.4)	1.000	0.04
Tricuspid regurgitation ≥2 +, *n* (%)	3 (5.7)	0 (0.0)	0.244	0.35
IVSd, cm	1.72 ± 0.46	1.99 ± 0.61	0.014	0.50
LVEDd, cm	4.20 ± 0.51	4.14 ± 0.58	0.605	0.10
LVPWd, cm	1.13 ± 0.18	1.33 ± 0.59	0.031	0.45
LA, cm	4.31 ± 0.72	4.27 ± 0.56	0.764	0.06
LVEF, %	68.2 ± 6.4	67.7 ± 6.5	0.681	0.08
PASP, mmHg	32.8 ± 7.7	32.3 ± 8.6	0.822	0.06
Maximum LV wall thickness, cm	2.25 ± 0.53	2.34 ± 0.52	0.389	0.17
LVOT-PGmax, mmHg	82.3 ± 46.0	77.9 ± 35.7	0.592	0.11
**Pre-PIMSRA medication use, *n* (%)**				
β-blocker, *n* (%)	48 (90.6)	46 (93.9)	0.717	0.12
Nondihydropyridine calcium-channel blocker, *n* (%)	6 (11.3)	7 (14.3)	0.770	0.09
Diuretic, *n* (%)	2 (3.8)	2 (4.1)	1.000	0.02
ACE inhibitor/ARB/ARNI, *n* (%)	9 (17.0)	11 (22.4)	0.619	0.14
Antiplatelet therapy, *n* (%)	3 (5.7)	1 (2.0)	0.619	0.19
Oral anticoagulant, *n* (%)	1 (1.9)	1 (2.0)	1.000	0.01

Values are mean ± SD or *n* (%). Pre-PIMSRA medication use was defined as medication documented as ongoing and not withheld during the 24 h preceding PIMSRA. ERAS, enhanced recovery after surgery; IVSd, interventricular septal thickness at end-diastole; LA, left atrial diameter; LVEDd, left ventricular end-diastolic dimension; LVPWd, left ventricular posterior wall thickness at end-diastole; LVEF, left ventricular ejection fraction; NYHA, New York Heart Association; PASP, pulmonary artery systolic pressure; LVOT-PGmax, maximum left ventricular outflow tract pressure gradient; PCI, percutaneous coronary intervention; ACE, angiotensin-converting enzyme; ARB, angiotensin receptor blocker; ARNI, angiotensin receptor-neprilysin inhibitor; PIMSRA, percutaneous intramyocardial septal radiofrequency ablation; bpm, beats per minute; LV, left ventricular; SD, standard deviation.

**Table 4:** ICU and hospitalization outcomes.

Outcome	Usual-Care Group	ERAS-Informed Group	Hodges–Lehmann Difference (95% CI)	*p* Value
Admission-to-PIMSRA interval, d	3.0 (2.0, 7.0)	3.0 (2.0, 5.0)	0.0 (–1.02 to 1.01)	0.309
ICU length of stay, h	23.0 (17.1, 45.5)	20.0 (5.8, 26.6)	−6.0 (−16.51 to −0.53)	0.022
Post-PIMSRA length of stay, d	6.0 (5.0, 6.0)	5.0 (5.0, 6.0)	0.0 (−1.04 to 0.01)	0.058
Total hospital stay, d	9.0 (8.0, 13.0)	8.0 (7.0, 11.0)	−1.0 (−2.02 to −0.03)	0.029

Values are medians (interquartile ranges). Hodges–Lehmann differences are expressed as ERAS-informed minus usual care. ICU, intensive care unit; ERAS, enhanced recovery after surgery; CI, confidence interval; PIMSRA, percutaneous intramyocardial septal radiofrequency ablation.

**Table 5:** Exploratory post hoc models for log-transformed hospitalization outcomes.

Outcome	exp(β) for log(LOS + 1) (95% CI)	Model-Based *p* Value
ICU length of stay, h	0.51 (0.34 to 0.76)	0.001
Post-PIMSRA length of stay, d	0.88 (0.79 to 0.99)	0.029
Total hospital stay, d	0.83 (0.72 to 0.95)	0.006

Exponentiated coefficients are from multivariable linear regression models for log(LOS + 1). Models adjusted for age, sex, baseline NYHA class (ordinal), IVSd, and calendar time, expressed as months from July 2023. The exp(β) estimate represents the ratio of the adjusted geometric means of LOS + 1 for the ERAS-informed group relative to the usual-care group; it is not a ratio of the arithmetic means or medians of the original LOS outcome. The models were post hoc and exploratory. No adjustment for multiple comparisons was applied; therefore, the confidence intervals and *p* values should be interpreted as hypothesis-generating. Because the timing of rehabilitation initiation was unavailable, these models could not address potential time-dependent selection or reverse causation. CI, confidence interval; LOS, length of stay; ERAS, enhanced recovery after surgery; ICU, intensive care unit; IVSd, interventricular septal thickness at end-diastole; NYHA, New York Heart Association; PIMSRA, percutaneous intramyocardial septal radiofrequency ablation.

**Table 6:** Echocardiographic parameters at baseline and discharge.

Parameter (Paired *n*: Usual-Care/ ERAS-Informed)	Usual-Care Baseline	Usual-Care Discharge	Within-Group *p*	ERAS-Informed Baseline	ERAS-Informed Discharge	Within-Group *p*	Between-Group Difference in Mean Change (95% CI)	*p* Value
IVSd, cm (49/49)	1.72 ± 0.47	1.73 ± 0.48	0.804	1.99 ± 0.61	1.92 ± 0.48	0.268	−0.08 (−0.23 to 0.08)	0.310
LVEDd, cm (49/49)	4.21 ± 0.50	4.18 ± 0.50	0.734	4.14 ± 0.58	4.32 ± 0.57	0.031	0.20 (−0.02 to 0.42)	0.073
LVPWd, cm (49/49)	1.13 ± 0.18	1.19 ± 0.23	0.071	1.33 ± 0.59	1.22 ± 0.29	0.158	−0.16 (−0.33 to 0.00)	0.052
LA, cm (49/49)	4.24 ± 0.60	4.28 ± 0.70	0.573	4.27 ± 0.56	4.17 ± 0.51	0.154	−0.15 (−0.36 to 0.06)	0.160
LVEF, % (49/49)	68.3 ± 6.5	64.0 ± 6.1	<0.001	67.7 ± 6.5	64.5 ± 5.5	0.015	1.11 (−2.19 to 4.41)	0.506
PASP, mmHg (19/26)	31.8 ± 7.2	29.6 ± 10.5	0.193	33.5 ± 8.3	32.7 ± 10.5	0.680	1.38 (−4.07 to 6.82)	0.612

Values are mean ± SD. Within-group changes were tested using paired *t* tests. Between-group differences in change were calculated as ERAS-informed minus usual care and tested using Welch independent-samples *t* tests. Change was calculated as discharge minus baseline. PASP analyses used available paired data. CI, confidence interval. Other abbreviations are defined in Table 3. For each parameter, baseline and discharge summaries, within-group comparisons, and between-group comparisons of change were based on the same patients with both measurements available.

## Data Availability

The datasets analyzed during the current study are not publicly available because they contain clinical data but may be available from the corresponding author on reasonable request and subject to appropriate institutional and ethics approval.
